# Evaluation of an Emergency Bulk Chlorination Project Targeting Drinking Water Vendors in Cholera-Affected Wards of Dar es Salaam and Morogoro, Tanzania

**DOI:** 10.4269/ajtmh.18-0734

**Published:** 2019-04-22

**Authors:** Anu Rajasingham, Colleen Hardy, Stanislaus Kamwaga, Kiwe Sebunya, Khalid Massa, Jane Mulungu, Andrea Martinsen, Evalyne Nyasani, Erin Hulland, Steven Russell, Curtis Blanton, Benjamin Nygren, Rachel Eidex, Thomas Handzel

**Affiliations:** 1 Emergency Response and Recovery Branch, Division of Global Health Protection, U.S. Centers for Disease Control and Prevention, Atlanta, Georgia;; 2 United Nations Children’s Fund Tanzania, Dar es Salaam, Tanzania;; 3 Tanzanian Ministry of Health, Community Development, Gender, Elderly and Children, Dar es Salaam, Tanzania;; 4 Global Immunizations Division, U.S. Centers for Disease Control and Prevention, Atlanta, Georgia;; 5 Tanzania Country Office, U.S. Centers for Disease Control and Prevention, Dar es Salaam, Tanzania

## Abstract

In August 2015, an outbreak of cholera was reported in Tanzania. In cholera-affected areas of urban Dar es Salaam and Morogoro, many households obtained drinking water from vendors, who sold water from tanks ranging in volume from 1,000 to 20,000 L. Water supplied by vendors was not adequately chlorinated. The Tanzanian Ministry of Health, Community Development, Gender, Elderly and Children and the U.N. Children’s Fund, Tanzania, collaborated to enroll and train vendors to treat their water with 8.68-g sodium dichloroisocyanurate tablets (Medentech, Ireland). The Centers for Disease Control and Prevention (CDC) provided monitoring and evaluation support. Vendors were provided a 3-month supply of chlorine tablets. A baseline assessment and routine monitoring were conducted by ward environmental health officers. Approximately 3 months after chlorine tablet distribution, an evaluation of the program was conducted. The evaluation included a full enumeration of all vendors, an in-depth survey with half of the vendors enumerated, and focus group discussions. In total, 797 (88.9%) vendors were included in the full enumeration and 392 in the in-depth survey. Free residual chlorine (FRC) was detected in 12.0% of tanks at baseline and 69.6% of tanks during the evaluation; however, only 17.4% of these tanks had FRC ≥ 0.5 mg/L. The results suggest high acceptability and use of the chlorine tablets by water vendors. However, given variation in the water source used and longer storage times, dosing could be increased in future programming. Bulk chlorination using chlorine tablets offers an efficient community-level approach to treating water closer to the point of use.

## INTRODUCTION

The Ministry of Health, Community Development, Gender, Elderly and Children (MoHCDGEC) of Tanzania first reported an outbreak of cholera in Dar es Salam region in August 2015.^1^ By February 2016, the outbreak had affected 22 of 25 mainland regions in Tanzania and resulted in 16,521 cases and 251 deaths. Urban areas of Dar es Salaam and Morogoro were heavily affected. Dar es Salaam reported 4,714 cases (29% of the mainland total), and Morogoro reported 1,325 cases (8% of the mainland total).^2^

Provision of safe drinking water is essential during cholera outbreaks, and chlorination of drinking water supplies is a high priority in both urban and rural settings. During a cholera outbreak, the World Health Organization (WHO) recommends free residual chlorine (FRC) levels of at least 0.5 mg/L at the point of use, 1.0 mg/L at tap stands, and 2.0 mg/L at tanker truck filling.^3^ In addition, routine monitoring of FRC levels is important to ensure the continued provision of safe water.^4^ Recent systematic reviews of water, sanitation, and hygiene (WASH) responses during cholera outbreaks and emergencies highlighted that water quality interventions implemented during outbreaks and emergencies have not been well documented.^5–7^ Distribution of chlorine-based household water treatment products is common during cholera outbreaks, and has been shown to improve the microbiological quality of water when paired with appropriate training and follow-up by community health workers.^8,9^ Community-level chlorination interventions implemented during cholera outbreaks are not well documented in the literature, with the exception of well chlorination programs.^6,10–13^

At the start of the outbreak, the municipal drinking water utilities in Dar es Salaam and Morogoro estimated that only 8–20% of the residents had in-home piped water connections.^14^ The remainder of residents obtained drinking water from a variety of sources, including private water vendors who sold water from large tanks that ranged in volume from 1,000 to 20,000 L. Vendors sold water in 20-L increments to nearby community members. Vendors sold water piped directly from the utility, water pumped from private boreholes, or water delivered by water trucks. The water sources for the trucks varied. Some collected water at utility truck filling stations, whereas others collected from other sources such as boreholes. In addition, some residents collected water from boreholes, shallow hand-dug wells, and rainwater.

Multiple challenges compromised the drinking water supply in both Dar es Salaam and Morogoro. First, although water supplied by the utilities was chlorinated, FRC levels throughout the piped network were inconsistent. Second, vendor tanks and trucks that filled from boreholes were not chlorinated. Third, trucks that filled from utility filling stations did not receive booster chlorination doses. At the peak of the outbreak and at the baseline of this program, spot checks from the piped water system, water trucks, and vendor tanks in Dar es Salaam resulted in detection of low levels of FRC. Free residual chlorine was detected in 36% (12/33) of samples from the piped water system, 53% (10/19) of samples from water trucks, and 12% (32/266) of samples from vendor tanks. Of the 32 samples from vendor tanks with detectable FRC, 24 samples were from tanks that filled from the piped water system and eight samples were from tanks that filled from water trucks. Morogoro district had similar issues with the piped utility and borehole water.

In response to the chlorination challenges observed in Dar es Salaam, Morogoro, and other regions, the government of Tanzania and partners took several steps: first, advocacy to municipal water utilities to increase chlorine levels to standards recommended during cholera outbreaks; second, strengthening the monitoring of the municipal distribution systems; third, distribution of household water treatment products to households in cholera hot spots; fourth, closure of shallow hand-dug wells; and fifth, social mobilization activities. However, few steps were taken to address the insufficient levels of chlorine in bulk drinking water supplies sold by private water vendors. The emergency bulk chlorination program targeting vendors in cholera-affected wards in Dar es Salaam and Morogoro aimed to address this gap, and started with a small pilot test in February 2016. Promising results led to expansion of the program.^15^

The emergency bulk chlorination program was a collaborative project between the U.N. Children’s Fund (UNICEF), Tanzania, the MoHCDGEC of Tanzania, and the US Centers for Disease Control and Prevention (CDC). MoHCDGEC and UNICEF implemented the program, and the CDC set up the monitoring and evaluation system. The program targeted large-volume water vendors in the 15 wards of Dar es Salaam and eight wards of Morogoro with the highest cholera attack rates at the start of the 2015 outbreak.

At the start of the program, ward environmental health officers were trained to identify, map, and enroll vendors with tanks of at least 1,000 L in volume into the bulk chlorination project. Ward officers conducted a baseline assessment, and vendors were invited to an orientation where they received project information and dosing instructions for the 8.68-g sodium dichloroisocyanurate (NaDCC) tablets (herein referred to as chlorine tablets), based on the specific volume of their tank(s). Each tablet contains 5 g of available chlorine, and the applied dose was approximately 1.0 mg/L. During pilot testing, this dose resulted in an FRC level of approximately 0.7 mg/L, 30 minutes after treatment. Vendors were trained to place tablets in tanks during or before tank filling to ensure proper mixing. Ward officers assigned to each of the targeted wards distributed the chlorine tablets to vendors and conducted routine monitoring visits at vendor locations throughout the 3-month program. Each vendor who continued in the program received at least a 3-month supply of chlorine tablets free of charge. In addition, the project allowed any non-mapped vendors or institutions in these wards, who expressed interest, to enroll at any time. In the fall of 2016, approximately 3 months after the first chlorine tablet distribution, the bulk chlorination program was evaluated in all implementation wards of Dar es Salaam and Morogoro.

## METHODS

### Evaluation design.

The evaluation included both quantitative and qualitative components: 1) a full enumeration of all mapped or enrolled vendors in the program, which included water quality testing and direct observations; 2) an in-depth survey with half of all vendors enumerated assessing vendor and customer preferences; and 3) focus group discussions (FGDs) with enrolled vendors, noncompliant vendors, defined as those who dropped out of the program, ward environmental health officers, and customers to identify strengths and weaknesses of the program. Combined, these components provided data on program implementation, chlorine tablet use, and vendor and customer acceptance.

### Quantitative methods.

We conducted a full enumeration of the 897 mapped or enrolled vendors in the bulk chlorination program (herein referred to as a census) using a short questionnaire. The short questionnaire included questions on vendor tanks, program participation, and reported treatment. In addition, the short questionnaire included direct observations and water testing where one tank from each vendor location was tested for FRC. At vendor locations where water was sold from multiple tanks, we prioritized testing for FRC in tanks that contained water for drinking and had water available at the time of the visit. FRC was tested using the N,N-diethyl-phenylenediamine colorimetric method using Hach CN66 test kits (Hach Co., Loveland, CO). In addition to the census, approximately 50% of all vendors, or every other vendor, were also interviewed using a longer, more comprehensive in-depth questionnaire, which included questions on vendor and customer preferences. Both questionnaires were translated, back-translated, and piloted before data collection. If a vendor could not be interviewed at the first visit, two additional attempts were made to interview each vendor.

### Qualitative data collection.

We aimed to conduct a total of 12 FGDs, three with each of the four targeted groups: 1) enrolled vendors; 2) non-compliant vendors, defined as those who dropped out of the program; 3) ward environmental health officers; and 4) customers. The objectives of the FGDs were to identify common themes and gain a better understanding of challenges related to the bulk chlorination program. The information collected during these discussions included in-depth details and variations that could not be captured during the quantitative surveys. A gender-balanced team of three Swahili/English speakers was trained over a 2-day period to conduct the FGDs. One leader facilitated the discussions using a prepared guide to ensure the inclusion of specific topics, and two others took detailed notes in Swahili during all discussions. FGDs were not time limited and continued until the facilitator felt the group reached saturation, meaning that no new or relevant information was raised. The team held a debrief following each FGD, and all Swahili notes were translated into English. All FGDs included both male and female participants.

### Ethical considerations.

The inclusion criterion for this evaluation was individuals (aged 18 years or older) who were responsible for water treatment at vendor tanks enrolled or mapped in the bulk chlorination program. The inclusion criterion for the ward officer and customer FGDs was officers or customers who were assigned to or lived in a ward where the bulk chlorination program was implemented. All FGD participants were older than 18 years. The CDC, Atlanta, submitted the evaluation protocol to the CDC’s Institutional Review Board (IRB), and it was classified as a non-research program evaluation (CGH protocol # 2016-214). The CDC, Tanzania, submitted the protocol to the Tanzania National Institute for Medical Research who granted IRB approval in September 2016.

### Data collection and analysis.

All survey data collection and entry were carried out on Samsung Galaxy tablets, using the open-source software Open Data Kit version 1.4.^16^ Team supervisors downloaded and reviewed data daily to identify any potential problems before the next day of data collection. Data analysis was conducted using SAS 9.4 (SAS Institute, Cary, NC) and RStudio 2017 (RStudio, Boston, MA). We described vendor use and acceptability of the chlorine tablets, program monitoring, and water treatment attitudes and practices descriptively using proportions. To obtain count data for values less than 1.0 mg/L, we multiplied all FRC values by 10. We then performed univariable and multivariable analyses using quasi-Poisson regression models to determine which factors were associated with detecting higher levels of FRC. The quasi-Poisson model was selected to account for the overdispersion of the FRC data, which was assessed using the dispersiontest function in RStudio.^17,18^ All variables significant at the *P* = 0.1 level in the univariable models were included into the multivariable model. Backward selection was used in the multivariable model to determine which parameters remained significantly related to FRC levels after controlling for other covariables; a selection criterion of *P* = 0.1 was used. Sensitivity analyses were performed using other parametrizations, including Poisson, zero-inflated Poisson, linear regression, and negative binomial regression.

All FGDs were transcribed from notes in Swahili, translated into English, coded, and organized by theme and frequency based on the questionnaire guidance document. Qualitative data were further broken down to verify census and in-depth survey data and identify new emergent themes.

## RESULTS

### Vendor survey demographics.

We visited all 897 vendors mapped or enrolled in the bulk chlorination program. In total, 797 (88.9%) vendors were available for the census survey: 666 in Dar es Salaam and 131 in Morogoro. Of these 797 vendors, 392 (49.2%) vendors were also included in the in-depth survey. In total, 60 of the 797 tank locations were at institutional settings: 43 at schools and 17 at mosques.

Survey participants were asked to self-identify as the tank owner, water seller, or both. Among those interviewed, 186 (23.3%) identified themselves as the owner, 328 (41.2%) as the seller, and 283 (35.5%) as being both the owner and seller. Approximately half of the interviewees (53.3%) were male with a median age of 39 years (range 18–80 years). Eight individuals were not interviewed because they were under the age criteria. The vendor survey results described in this report combine the results from Dar es Salaam and Morogoro. Data collected from the census and in-depth survey are described separately.

#### Census survey.

##### Vendor sales.

As part of the census survey, vendors were asked about water sales; 647/797 (81.2%) vendors reported selling water in the previous 7 days. The majority of vendors, 418/647 (64.6%), reported selling from one tank (range 1–7 tanks), 160/647 (24.7%) reported selling from two tanks, and 65/647 (10.0%) reported selling from three or more tanks. The reasons given for not currently selling water included unavailability of water, broken tank or pump, not a water vendor, institutional use (school or mosque use), or water provided for free.

##### Vendor training and orientation.

Vendors were asked about their reasons for joining the bulk chlorination program. The most commonly cited responses were “wanted to sell safe water” (465/797; 58.3%) and “to prevent diseases” (457; 57.3%). When asked about participation in any training or orientation before joining the bulk chlorination program, 752 (94.4%) vendors reported receiving training on water treatment using the chlorine tablets and 776 (97.4%) reported receiving chlorine tablets from their ward environmental health officers. The most common reason cited for not receiving chlorine tablets was that vendors were unaware of the orientation in their ward (6/17; 35.3%).

##### Water testing.

As part of the census, one tank from all vendor locations was selected for FRC testing; 756/797 (94.9%) vendors had working tanks available for this selection. Of these tanks, 94.4% were situated under direct sunlight. Water was available for sampling at 675 (89.3%) of the vendors visited. The median tank size was 5,000 L (range 1,000–20,000 L), and 71.3% of water tested was sold primarily as drinking water. The majority of water samples tested, 335 (49.6%), originated from boreholes. Almost half of the tanks tested, 334 (49.5%), were elevated, which we defined as being at least 3 m above ground level. Of the 334 elevated tanks tested, 293 (87.7%) contained borehole water.

All vendors who had water available for testing were asked about tank treatment. In total, 488/675 (72.3%) reported treating tank water at a median time of 24 hours ago (range < 1 hour–2 weeks). The FRC levels detected ranged from 0 to 3.4 mg/L, and the mean FRC detected was 0.2 mg/L (median 0.1 mg/L). Free residual chlorine was detected in 470/675 (69.6%) of all tanks tested (Table 1). Moreover, FRC was detected in 402/488 (82.4%) tanks where vendors reported treating the water in their tank, but only 17.4% of these tanks had FRC ≥ 0.5 mg/L. Of note, 86/488 (17.6%) of those tanks where vendors reported treatment had no detectable FRC (Table 1). Among vendors who provided specific information on the number of chlorine tablets added to their tank, 393/445 (88.3%) applied the appropriate dose, which was determined by comparing the number of reported chlorine tablets added to the observed volume of their tank.

**Table 1:** Free residual chlorine results from all tanks tested versus tanks with reported treatment

Free residual chlorine (mg/L)	All tanks tested, *N* = 675	Tanks with reported treatment, *N* = 488
	*n* (%)	*n* (%)
0	205 (30.4)	86 (17.6)
0.1–0.4	380 (56.3)	317 (65.0)
0.5–0.9	53 (7.8)	50 (10.2)
≥ 1.0	37 (5.5)	35 (7.2)

#### In-depth survey.

##### Vendor training, chlorine tablet distribution, and monitoring.

The 392 vendors who were included in the in-depth survey were asked additional questions about any training they received, chlorine tablet distribution, and monitoring by ward environmental health officers. Of these 392 vendors, 328 (83.8%) vendors reported attending an orientation facilitated by their ward environmental health officers. At the orientation, 317 (96.7%) vendors reported receipt of dosing instructions, and 59 (18.0%) obtained information on how to receive additional chlorine tablets.

The majority of vendors, 317/328 (96.7%), who participated in the in-depth survey and attended an orientation reported receiving chlorine tablets at the orientation led by ward environmental health officers. However, 86/392 (21.9%) vendors surveyed reported receiving chlorine tablets in other ways. Of these, 70.9% reported that ward officers delivered chlorine tablets to their location, whereas 34.9% retrieved them at the ward office. The majority of vendors, 302 (77.0%), reported having remaining stock of chlorine tablets, and when asked to show what remained, 298 (76.0%) vendors were able to show the tablets. Among those vendors with remaining chlorine tablets, the median number of tablets was 13 tablets (range 0–180 tablets).

The majority of vendors, 348 (88.8%), who participated in the in-depth survey reported program monitoring visits by ward environmental health officers. Among those visited, the median number of reported visits since program initiation was three (range 1–25 visits). During monitoring visits, 77.6% of vendors reported that the ward officer always tested for FRC.

##### Vendor and customer preferences.

As part of the in-depth survey, we asked vendors about their perceptions of the bulk chlorination program. We asked vendors about the ease or difficulty of treating water in their tanks. The majority, 351/392 (89.5%), found it easy to treat with chlorine tablets, 22/392 (5.6%) found it difficult, 3.1% did not know, and 1.8% felt it was neither easy nor difficult. Among the 22 who found it difficult to treat, 90.9% stated it was the tank location that made it difficult (e.g., tank on roof) and 9.1% said it was difficult to break the chlorine tablets in half or quarters to treat tanks with volumes less than 5,000 L.

The majority of vendors included in the in-depth survey, 316 (80.6%), reported willingness to purchase chlorine tablets if made available for sale on the market. Vendors suggested a number of potential selling locations: pharmacies (57.8%), ward health offices (29.5%), sub-ward chairman’s offices (39.2%), shops (15.9%), or health facilities (7.9%).

During the in-depth survey, we also asked vendors about their perceptions of customers’ preferences. The majority of vendors, 317/392 (80.9%), felt customers preferred chlorinated water. When asked about customer complaints, 79/392 (20.2%) vendors stated that customers complained about the chlorinated water. Of these, 88.6% of the vendors reported customer complaints on the smell of water, 26.6% on the taste of water, and 6.3% on the color of water.

##### *Univariable analyses*.

Univariable analyses using quasi-Poisson models were performed to determine which factors were associated with detecting higher levels of FRC. The quasi-Poisson model was selected as the dispersiontest function in R demonstrated that the FRC data were significantly overdispersed (*P* = 0.0077). The factors significantly associated with higher levels of FRC in vendor tanks included receiving training on the tablets (3.21 [1.07–9.67], *P* = 0.039), using piped or truck water versus borehole water (1.53 [1.16–2.02], *P* = 0.003), and shorter time since treatment and storage: less than 24 hours (3.84 [2.41–6.11], *P* ≤ 0.0001), between 25 and 72 hours (2.89 [1.70–4.89], *P* ≤ 0.0001), or 72 or more hours (1.73 [0.91–3.29], *P* ≤ 0.0001) (Table 2). Tanks under sunlight (0.52 [0.34–0.80], *P* = 0.003) were associated with lower levels of FRC, and having an elevated tank (0.79 [0.60–1.03], *P* = 0.077) approached significance (Table 2).

**Table 2:** Univariable quasi-Poisson regression estimates

Variable	*P*-value	Estimate (95% CI)
Received training on chlorine tablets	0.039	3.21 (1.07–9.67)
Yes		Ref
No		
Piped or trucked water	0.003	1.53 (1.16–2.02)
Yes		Ref
No		
Time since treatment (hours)	< 0.0001	3.84 (2.41–6.11)
≤ 24		2.89 (1.70–4.89)
25–72		1.73 (0.91–3.29)
72+		Ref
No treatment		
Tank under sunlight	0.003	0.52 (0.34–0.80)
Yes		Ref
No		
Elevated tank	0.077	0.79 (0.60–1.03)
Yes		Ref
No		

##### *Multivariable analyses*.

Multivariable analyses using backward selection included all factors significant in the univariable model (Table 3). The factors significantly associated with detecting higher levels of FRC in vendor tanks using a quasi-Poisson model included using piped or truck water (1.82 [1.34–2.49], *P* = 0.002) and shorter time since treatment: less than 24 hours (4.11 [2.48–6.79], *P* ≤ 0.0001), between 25 and 72 hours (2.54 [1.43–4.50], *P* ≤ 0.0001), or 72 or more hours (1.46 [0.73–2.94], *P* ≤ 0.0001). Tanks in direct sunlight were significantly associated with lower levels of FRC (0.61 [0.38–0.99], *P* = 0.047). We conducted a sub-analysis using data from vendors using only borehole water to understand whether any potential chlorination by the utility affected results. In these analyses, our power was limited by sample size, as only 327 met the criteria for this sub-analysis. Although variables dropped out of this secondary model in the same order as the primary model (gender, age, elevated tank, sells water, received training, and sunlight), the only significant factor that remained in the model of vendors using only boreholes was shorter time since treatment.

**Table 3:** Multivariable quasi-Poisson regression estimates

Variable	*P*-value	Estimate (95% CI)
Piped or trucked water	0.002	1.82 (1.34–2.49)
Yes		Ref
No		
Tank under sunlight	0.047	0.61 (0.38–0.99)
Yes		Ref
No		
Time since treatment (hours)	< 0.0001	4.11 (2.48–6.79)
≤ 24		2.54 (1.43–4.50)
25–72		1.46 (0.73–2.94)
72+		Ref
No treatment		

##### *Qualitative data*.

Eight of the 12 planned FGDs were conducted with enrolled vendors, ward environmental health officers, and customers (Table 4). Participants in specific focus groups consisted of 23 ward officers, 12 customers, and 24 vendors. All FGDs included both males and females, and in total, 35 males and 24 females participated. Ages ranged from 22 to 80 years. On average, discussions lasted 1.4 hours with a range of 37 minutes to 2 hours and 6 minutes. The longest discussions were among the ward environmental health officers, which lasted more than 2 hours.

**Table 4:** Focus group discussions by target group and location

Location	Vendors enrolled		Vendors noncompliant/not treating		Ward officers		Customers	
No. of discussions	Planned	Actual	Planned	Actual	Planned	Actual	Planned	Actual
Dar es Salaam	2	2	2	0	2	2	2	1
Morogoro	1	1	1	0	1	1	1	1
Total groups	3	3	3	0	3	3	3	2

Themes identified from the FGDs supported findings from the in-depth survey on the importance of ward officer monitoring and challenges associated with treating elevated tanks. Vendor FGDs highlighted the importance of ward officer monitoring. Vendors stated that officers were always available for advice and made regular visits, and that their involvement signified that the government was aware of and supported the program. Vendors felt that local government involvement gave them confidence to participate in the program. During both vendor and ward officer FGDs, elevated tanks were highlighted as a barrier to treatment. Additional equipment such as a step ladder was required, and extra assistance was necessary for elderly or disabled vendors unable to climb to the top of the tanks. One of the participants said, “I always struggle to treat my water because my tank is elevated and I always ask for help from someone.”

Focus group discussions revealed that vendors and customers recognized the importance of drinking safe water during a cholera outbreak, and as a result, vendors were motivated to sell safe water. Vendors noted that treating water increased sales. One vendor said, “Chlorine tables make the water safe for consumption, which has increased the number of customers.” Both vendors and customers revealed high knowledge of the benefits and importance of safe water during FGDs; these were motivators for vendors to provide safe water and for customers to purchase safe water from vendors. One customer stated, “I see vendors adding tablets to the tanks most of the time, so I trust them.” Although the issues of taste and smell were raised during the customers’ FGDs, neither was raised as potential barriers to purchasing chlorinated water. One customer said, “There is a chlorine odor but the taste is ok. We are now used to the treated water and we immediately recognize untreated water because of the smell.”

## DISCUSSION

Approximately 3 months after the bulk chlorination project began, we detected FRC in approximately 70% of all vendor tanks tested and in more than 82% of tanks reported to have been treated. These results suggest high acceptability and use of the chlorine tablets by vendors in Dar es Salaam and Morogoro. In addition, these results indicate that vendors changed their behavior to treat tank water. Bulk chlorination offers an efficient method to provide treated water to populations at risk of cholera, without having to rely on household water treatment options, which require additional resources to ensure water treatment behavior change.^19,20^ Bulk chlorination is also less labor intensive to distribute and monitor during emergencies, as fewer points need to be targeted and visited. Although bulk chlorination has been used in other settings and emergencies, to our knowledge, this is the first documented program where vendors were provided a means to chlorinate their water in bulk.

Although enrollment into the project and the use of chlorine tablets were optional, there are several reasons why reported use was high. First, all vendors in the targeted cholera-affected communities were mapped, visited individually, invited to join the program, and provided chlorine tablets for free, which reduced economic and logistical barriers to procuring and using the product. Second, as these areas were previously heavily affected by cholera, vendors cited wanting to sell safe water and to prevent diseases as the main reasons for using the chlorine tablets. This motivation was further supported during vendor FGDs. Third, only 21.5% of vendors reported running out of chlorine tablets at some point during the program, indicating that ward environmental health officers were able to distribute tablets to vendors in a timely manner. Fourth, program awareness among vendors was high, with chlorine tablets observed in 76.0% of locations and with 88.3% of vendors appropriately dosing their tanks. Fifth, 88.8% of vendors reported receiving a monitoring visit and the median number of visits reported was three over the course of 3 months; these monitoring visits likely reinforced messages on the importance of treatment.

Although 69.6% of all tested tanks (*n* = 675) had detectable FRC, only 17.4% of the tanks that were reported as treated (*n* = 488) had FRC ≥ 0.5 mg/L, the level recommended to prevent household contamination after water is purchased and stored in the home. Moreover, 17.6% of those that reported treating tank water had no FRC detected in their tanks. There are several possible explanations for the detection of low levels of FRC in vendor tanks. First, the applied dose for a 5,000-L tank was approximately 1.0 mg/L; based on pilot testing, this resulted in an FRC of approximately 0.7 mg/L, 30 minutes after treatment. The applied dose remained on the conservative side so that tanks that filled from potentially previously chlorinated sources such as piped or trucked water were not over-chlorinated. However, this dose may not have been sufficient for all water sources used in this program. Second, the median reported time since treatment found during the evaluation was 24 hours (range < 1 hour–2 weeks), and vendors stored water until depletion. Thus, FRC levels likely decreased over time, and, in some cases, remained detectable. A limitation of this bulk chlorination program was that it was difficult to maintain consistent FRC in tanks with long storage times, as each tank was treated once and vendors were unable to add booster doses. Third, the chlorine demand was not consistent across water sources used. The lower levels of FRC detected in borehole water versus piped or trucked water could be due to the higher chlorine demand of certain boreholes in this setting. We tested pH, conductivity, total dissolved solids (TDS), hardness, iron, and manganese in a small number of boreholes used and found that chlorine demand was higher in wells with TDS levels more than 1,000 ppm (data not shown). Additional research should be conducted to investigate the reasons for this higher chlorine demand. Fourth, 94.4% of tanks tested were located under direct sunlight, and tanks situated under direct sunlight were significantly associated with lower FRC levels. Higher temperatures of tanks located under direct sunlight may have contributed to FRC dissipation.^21^

Almost half of all tanks tested, 49.6%, were elevated, and treatment of elevated tanks was noted as a barrier to treatment during both the in-depth survey and FGDs. In some areas of Dar es Salaam, we observed modifications to tanks to allow for the treatment of elevated tanks. This included the addition of an extra valve to tanks above pumps. During tank filling, when the valve was opened and the pump turned on, water pressure brought the chlorine tablets up to elevated tanks. In addition, one alternative method piloted was installing a point-of-entry chlorinator, Aquatabs Flo^©^ (Medentech, Ireland), in vendor tanks, which provided an adjustable chlorine dose when the tank was filled.^22^ Pilot monitoring data indicated that the chlorinator provided a consistent dose; however, as chlorine cartridges need to be replaced, routine monitoring was important. Treatment of elevated tanks should be considered when implementing this intervention.

This bulk chlorination program was designed as an emergency intervention, and chlorine tablets were provided to vendors free of charge. When vendors needed additional chlorine tablets, they were able to pick up tablets at ward health offices, and in some cases, ward health officers delivered tablets to vendor locations. However, for bulk chlorination to be a long-term preventative intervention, locations where chlorine tablets are sold should be nearby and convenient. In addition, sustainable cost-sharing pricing would need to be in place for both vendors and customers to motivate purchases and water treatment. Social marketing may be required to educate customers on the benefits of purchasing treated water from vendors. If the market allows, additional tablet sizes (1.67 g or 2.5 g) could be sold for use in smaller volume tanks.

## LIMITATIONS

There were several limitations to this evaluation. First, there was likely recall and courtesy bias among vendors: recall bias as respondents may not have remembered when they last treated their tank, and courtesy bias as respondents may have understated any dissatisfaction with the product and over-reported treatment. Second, 64.5% of tanks were owned and operated by different people; therefore, there were multiple individuals involved in collecting tablet supplies and treating tanks and we may not have interviewed the most knowledgeable person. Third, we were unable to hold one of the planned three FGDs for water vendor customers, therefore limiting the information gathered for this group. Fourth, we were unable to recruit participants for the three planned FGDs for noncompliant vendors, preventing us from gathering qualitative information from vendors who dropped out of the program. Finally, this evaluation was cross-sectional; thus, we are not able to comment on the frequency of treatment over the duration of the program.

## CONCLUSION

The results from this evaluation indicate that bulk chlorination targeting medium- to large-volume water suppliers such as water vendors could be a feasible mechanism to address chlorination gaps at the community level during emergencies and disease outbreaks. This program provided a foundation for community-level water treatment by targeting vendors who have the ability to treat water at the point of collection before selling water to households in smaller volumes. This approach may complement chlorination efforts in areas where treatment occurs directly at the source and is followed by long transportation times or intermittent supply, factors that can contribute to decreased levels of FRC detected in piped municipal water.^23,24^ In addition, previous research has found that although household water treatment is proven to be effective in improving the microbial quality of water and reducing diarrheal disease outcomes, ensuring sustainable water treatment behavior change at the household level is challenging in both development and emergency settings.^8,25–30^ Bulk chlorination using large chlorine tablets offers a novel and more efficient way to chlorinate water closer to the point of use, without the challenges of changing behavior at the household level during an emergency. This approach could be used during other WASH emergencies or as a preventative measure to prevent transmission of diarrheal diseases in other low- and middle-income countries with similar chlorination and water storage challenges.

## References

[1] Narra R 2017 Notes from the field: ongoing cholera epidemic—Tanzania, 2015–2016. MMWR Morb Mortal Wkly Rep 66: 177–178. 28207686 10.15585/mmwr.mm6606a5 PMC5657858

[2] MoHCDGEC, 2016 Tanzania Cholera Situation Report February 23, 2016. Dar es Salaam, Tanzania: MoHCDGEC.

[3] WHO, 1993 Guidelines for Cholera Control. Geneva, Switzerland: World Health Organization Available at: http://www.cliniciansoftheworld.org/WHO_Guidelines_Cholera_Control.pdf. Accessed September 1, 2017.

[4] WHO, 2016 Guidelines for Drinking-Water Quality, 4th edition Geneva, Switzerland: World Health Organization Available at: http://apps.who.int/iris/bitstream/10665/44584/1/9789241548151_eng.pdf. Accessed November 5, 2016.

[5] Ramesh A Blanchet K Ensink JHJ Roberts B, 2015 Evidence on the effectiveness of water, sanitation, and hygiene (WASH) interventions on health outcomes in humanitarian crises: a systematic review. PLoS One 10: e0124688. 26398228 10.1371/journal.pone.0124688 PMC4580573

[6] Taylor DL Kahawita TM Cairncross S Ensink JHJ, 2015 The impact of water, sanitation and hygiene interventions to control cholera: a systematic review. PLoS One 10: e0135676. 26284367 10.1371/journal.pone.0135676 PMC4540465

[7] Branz A Levine M Lehmann L Bastable A Imran Ali S Kadir K Yates T Bloom D Lantagne D, 2017 Chlorination of drinking water in emergencies: a review of knowledge, recommendations for implementation, and research needed. Waterlines 36: 4–39.

[8] Lantagne D Yates T, 2018 Household water treatment and cholera control. J Infect Dis 218 (Suppl 3): S147–S153. 30215739 10.1093/infdis/jiy488 PMC6188534

[9] Lantagne DS Clasen TF, 2012 Use of household water treatment and safe storage methods in acute emergency response: case study results from Nepal, Indonesia, Kenya, and Haiti. Environ Sci Technol 46: 11352–11360. 22963031 10.1021/es301842u

[10] Clasen TF Alexander KT Sinclair D Boisson S Peletz R Chang HH Majorin F Cairncross S, 2015 Interventions to improve water quality for preventing diarrhoea. Cochrane Database Syst Rev 10: CD004794. 10.1002/14651858.CD004794.pub3. PMC4625648 26488938

[11] Cavallaro EC Harris JR da Goia MS dos Santos Barrado JC da Nobrega AA de Alvarenga de Junior IC Silva AP Sobel J Mintz E, 2011 Evaluation of pot-chlorination of wells during a cholera outbreak, Bissau, Guinea-Bissau, 2008. J Water Health 9: 394–402. 21942203 10.2166/wh.2011.122

[12] Garandeau R Trevett A Bastable A, 2006 Chlorination of hand-dug wells in Monrovia. Waterlines 24: 19–21.

[13] Guevart E Van Hecke C Noeske J Solle J Bita Fouda A Manga B, 2008 Diffuseur artisanal de chlore pour desinfecter les puits lors de l'epidemie de cholera de Douala. Med Trop 68: 507–513. 19068985

[14] UNDP, 2011 Services and Supply Chains: The Role of the Domestic Private Sector in Water Service Delivery in Tanzania. New York, NY: United Nations Development Programme Available at: http://www.undp.org/content/undp/en/home/librarypage/poverty-reduction/inclusive_development/services_and_supplychainstheroleofthedomesticprivatesectorinwate/. Accessed December 6, 2016.

[15] Wang A 2016 Notes from the field: chlorination strategies for drinking water during a cholera epidemic—Tanzania, 2016. MMWR Morb Mortal Wkly Rep 65: 1150–1151. 27764079 10.15585/mmwr.mm6541a6

[16] Brunette W Sundt M Dell N Chaudhri R Breit N Borriello G, 2013 Open Data Kit 2.0: Expanding and Refining Information Services for Developing Regions. 2013 HotMobile Workshop: 14th International Workshop on Mobile Computing Systems and Applications, Jekyll Island, GA Available at: http://www.hotmobile.org/2013/papers/full/2.pdf. Accessed September 5, 2017.

[17] Kleiber C Zeileis A, 2008 Applied Econometrics with R. New York, NY: Springer-Verlag ISBN 978-0-387-77316-2. Available at: https://CRAN.R-project.org/package=AER Accessed September 1, 2017.

[18] R Core Team, 2017 R: A Language and Environment for Statistical Computing. Vienna, Austria: R Foundation for Statistical Computing Available at: https://www.R-project.org/. Accessed September 2, 2017.

[19] Fiebelkorn AP Person B Quick RE Vindigni SM Jhung M Bowen A Riley PL, 2012 Systematic review of behavior change research on point-of-use water treatment interventions in countries categorized as low- to medium-development on the human development index. Soc Sci Med 75: 622–633. 22497845 10.1016/j.socscimed.2012.02.011

[20] Clasen TF, 2009 Scaling up Household Water Treatment Among Low-Income Populations. Geneva, Switzerland: World Health Organization Available at: http://www.wpro.who.int/vietnam/publications/scaling_up_hwts_in_low_income_community.pdf. Accessed November 5, 2017.

[21] Nouri A Shahmoradi B Dehestani-Athar S Maleki A, 2015 Effect of temperature on pH, turbidity, and residual free chlorine in Sanandaj water distribution network, Iran. J Adv Environ Health Res 3: 188–195.

[22] Medentech, 2017 *Aquatabs Flo*. Available at: http://www.aquatabs.com/home/product-range/aquatabs-flo/. Accessed February 2, 2017.

[23] Elala D Labhasetwar P Tyrrel S, 2011 Deterioration in water quality from supply chain to household and appropriate storage in the context of intermittent water supplies. Water Sci Technol Water Supply 11: 400–408.

[24] Kumpel E Nelson KL, 2013 Comparing microbial water quality in an intermittent and continuous piped water supply. Water Res 47: 5176–5188. 23866140 10.1016/j.watres.2013.05.058

[25] Clasen T Schmidt WP Rabie T Roberts I Cairncross S, 2007 Interventions to improve water quality for preventing diarrhoea: systematic review and meta-analysis. BMJ 334: 782. 17353208 10.1136/bmj.39118.489931.BE PMC1851994

[26] Crump JA Otieno PO Slutsker L Keswick BH Rosen DH Hoekstra M Vulule J Luby S, 2005 Household based treatment of drinking water with flocculant-disinfectant for preventing diarrhoea in areas with turbid source water in rural western Kenya: cluster randomised controlled trial. BMJ 331: 478. 16046440 10.1136/bmj.38512.618681.E0 PMC1199021

[27] Luby SP Agboatwalla M Hoekstra RM Rahbar MH Billhimer W Keswick BH, 2004 Delayed effectiveness of home-based interventions in reducing childhood diarrhea, Karachi, Pakistan. Am J Trop Med Hy 71: 420–427. 15516637

[28] Fewtrell L Kaufmann R Kay D Enanoria W, 2005 Water, sanitation, and hygiene interventions to reduce diarrhoea in less developed countries: a systematic review and meta-analysis. Lancet Infect Dis 5: 42–52. 15620560 10.1016/S1473-3099(04)01253-8

[29] Makutsa P Nzaku K Ogutu P Barasa P Ombeki S Mwaki A Quick R, 2001 Challenges in implementing a point-of-use water quality intervention in rural Kenya. Am J Public Health 91: 1571–1573. 11574308 10.2105/ajph.91.10.1571 PMC1446827

[30] Francis M Nagarajan G Sarkar R Mohan VR Kang G Balraj V, 2015 Perception of drinking water safety and factors influencing acceptance and sustainability of a water quality intervention in rural southern India. BMC Public Health 15: 731. 26223687 10.1186/s12889-015-1974-0 PMC4520261

